# Mapping research trends of retinal vein occlusion from 2009 to 2018: a bibliometric analysis

**DOI:** 10.7717/peerj.7603

**Published:** 2019-08-29

**Authors:** Fangkun Zhao, Fengkun Du, Dong Shi, Wenkai Zhou, Youhong Jiang, Liwei Ma

**Affiliations:** 1 Department of Ophthalmology, The Fourth Affiliated Hospital of China Medical University, Eye Hospital of China Medical University, Key Lens Laboratory of Liaoning Province, Shenyang, Liaoning, China; 2 Department of Biology, Xavier University of Louisiana, New Orleans, LA, United States of America; 3 Department of Molecular Oncology, Cancer Research Institution, The First Affiliated Hospital of China Medical University, Shenyang, China

**Keywords:** VOSviewer, Bibliometric analysis, Retinal vein occlusion

## Abstract

**Objectives:**

To map publication trends and explore research hotspots of retinal vein occlusion (RVO) study.

**Methods:**

Based on Web of Science Core Collection (WoSCC), a bibliometric analysis was carried out. The knowledge map was constructed by VOSviewer v.1.6.10 to visualize the annual publication number, the distribution of countries, international collaborations, author productivity, source journals, cited reference and keywords in this field.

**Results:**

A total of 2,135 peer-reviewed papers were retrieved on RVO from 2009 to 2018. The United States ranks highest among countries with the most publications and the most active institution was Kyoto University. Noma H contributed the most publications in this field. *Retina—The Journal of Retinal and Vitreous Disease* was the most prolific journal in RVO research. The top cited references mainly presented anti-VEGF medications on the management of RVO. The keywords formed six clusters: (1) Risk factors and pathogenesis of RVO; (2) Metabolismof RVO; (3) Therapeutic use of corticosteroids on RVO; (4) Diagnostic methodsof RVO; (5) Management of macular edema secondary to RVO (6) Anti-VEGFtreatment of RVO.

**Conclusions:**

The six major research hotspots could provide an insight into RVO research and valuable information for researchers to identify potential collaborators and partner institutions.

## Introduction

Retinal vein occlusion (RVO) is a retinal vascular disease that threatens the vision and associated with macular edema and neovascularization (Hahn & Fekrat, 2012). According to the occlusion site, RVO is mainly divided into central (CRVO) and branch (BRVO). A large number of research papers related to RVO have been published in academic journals since the past decades. In the present study, we applied the bibliometric methods and mapping knowledge domain (MKD) methods to explore the research status in RVO study.

Bibliometric analysis is a method to analyze relevant literature by mathematical statistics. The distribution, correlation and clustering of literatures can be measured quantitatively (Zou, Yue & Vu, 2018). With databases and visualization technology, the MKD method provides a new way to conduct literature mining and reveal the core structure of scientific knowledge. Recently, co-citation analyses and keyword co-occurrence analyses are utilized for knowledge mapping.

Specifically, this study assessed the growth in publications, international collaborations, author productivity, source journals, co-citation analysis and keyword co-occurrence analysis related to RVO study. Assessing research trends of an academic area are important for researchers to explore. Bibliometric hotspots analysis can act as a visual tool to evaluate important trends in research, as well as identify understudied areas of importance. Therefore, the purpose of our study is to conduct a comprehensive analysis of the scientific literatures related to RVO.

## Methods

### Data source and research process

The Science Citation Index Expanded database in the Web of Science Core Collection (WoSCC) was retrieved online as the source for the study. The retrieval keyword was “retinal vein occlusion”, the document type was “article”, and the time span was “from 2009 to 2018”. No language restrictions were set. The retrieved results were saved as “Plain text” with “full record and cited references”. The following basic information regarding each article was collected: country, author, institution, journal, references, and keywords.

### Analytical tool and method

Visualization software can generate node-link maps which can be used to visually observe the research distribution, hotspots, and direction of research development. In this study, the data were imported into VOSviewer v.1.6.10 and analyzed systematically. VOSviewer (http://www.vosviewer.com) developed by Van Eck & Waltman (2010), is a literature visualization software which has advantages of displaying cluster analysis results. In the knowledge maps generated by VOSviewer, items are represented as nodes and links. The nodes and their labels, such as countries, organizations, authors, co-citation literatures, and keywords, are proportional to the weight of the analysis components. Relationships between elements can be presented by links between the nodes.

In this study, co-citation cited reference and keyword co-occurrence networks were applied to construct the knowledge map of RVO study. Cluster analysis of similar co-cited cited reference could be used to summarize the main topics in the knowledge base. Keywords can express the theme of literatures, and clustering analysis of these co-occurrence keywords can reveal the knowledge structure and hotspots in this research field.

## Results

### Annual distribution of publications

**Figure 1:**
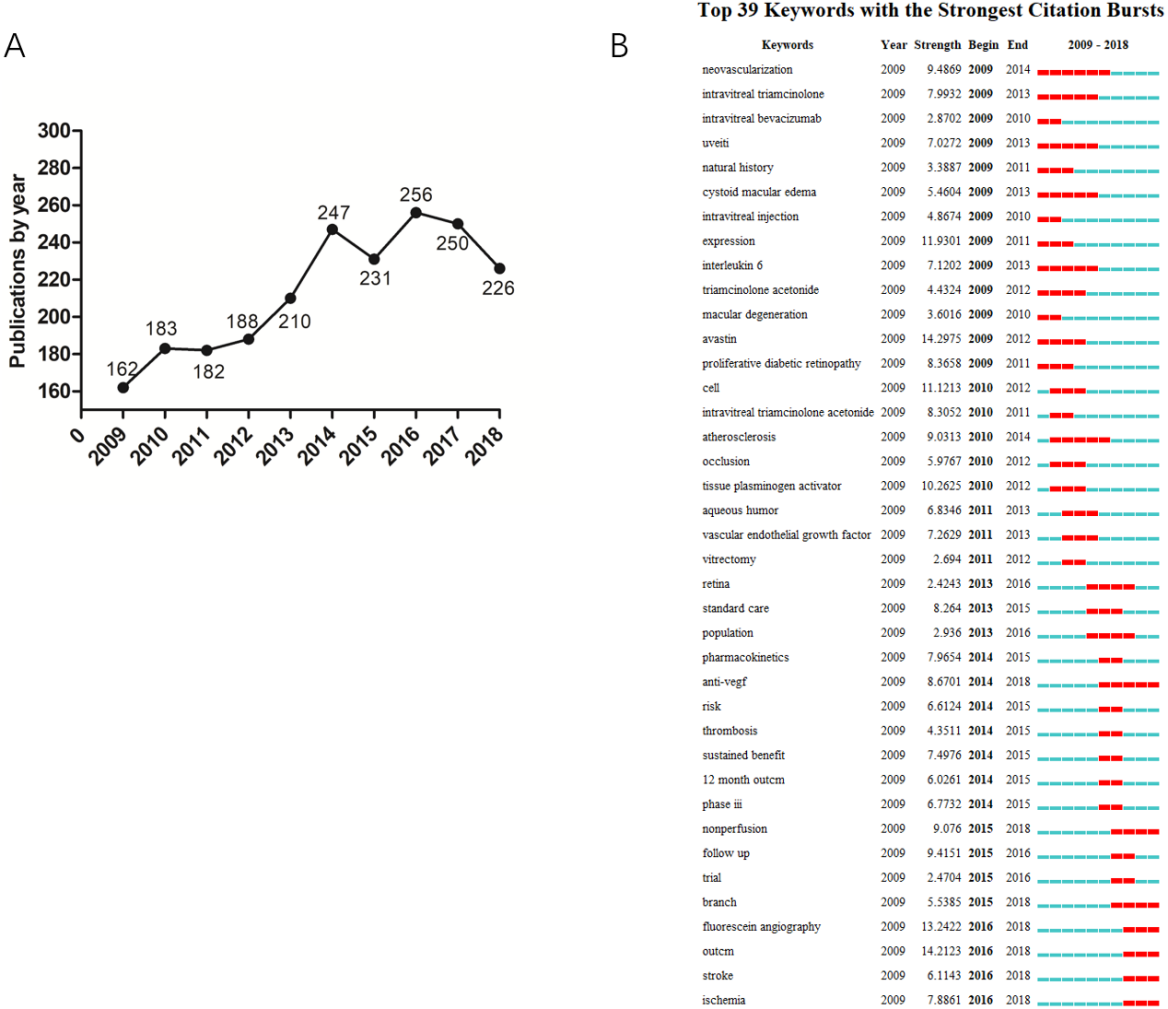
(A) The annual number of publications in RVO research from 2009 to 2018. (B) Burst analysis of keywords.

Based on the bibliometric retrieval results, WoSCC has collected 2,135 articles on RVO from 2009 to 2018. The number of published papers has arisen in general in the past decade which rose from 162 articles to 226 articles (Fig. 1A). Through keywords burst analysis, the top 39 keywords with the strongest citation bursts were extracted. Among these words “anti-VEGF” showed citation burst from 2014 (Fig. 1B), which is consistent with the boost of published papers.

### Country analysis

According to the retrieved results, the 2,135 articles originated from 75 countries. As presented in Table 1, the top 10 countries engaged in RVO research have published 1,908 articles, accounting for 89.4% of the total number of publications. The United States contributed the most publications (519, 24.3%), followed by Japan (300, 14.1%) and Germany (256, 12.0%). Based on citation analysis, the United States had 12,096 citations, followed by Japan (3,887 citations) and Germany (3,732 citations).

**Table 1:** Top 10 productive countries in RVO study, 2009–2018.

Rank	Country	Count (%)	Citations
1	USA	519 (24.3)	12,096
2	Japan	300 (14.1)	3,887
3	Germany	256 (12.0)	3,732
4	China	179 (8.4)	1,351
5	South Korea	142 (6.7)	2,064
6	Italy	121 (5.7)	2,168
7	England	106 (5.0)	1,386
8	Turkey	103 (4.8)	384
9	France	100 (4.7)	1,312
10	Switzerland	83 (3.8)	794

**Notes.**

Table percentages were calculated by dividing the row count by the total number of publications (*n* = 2,135).

**Figure 2:**
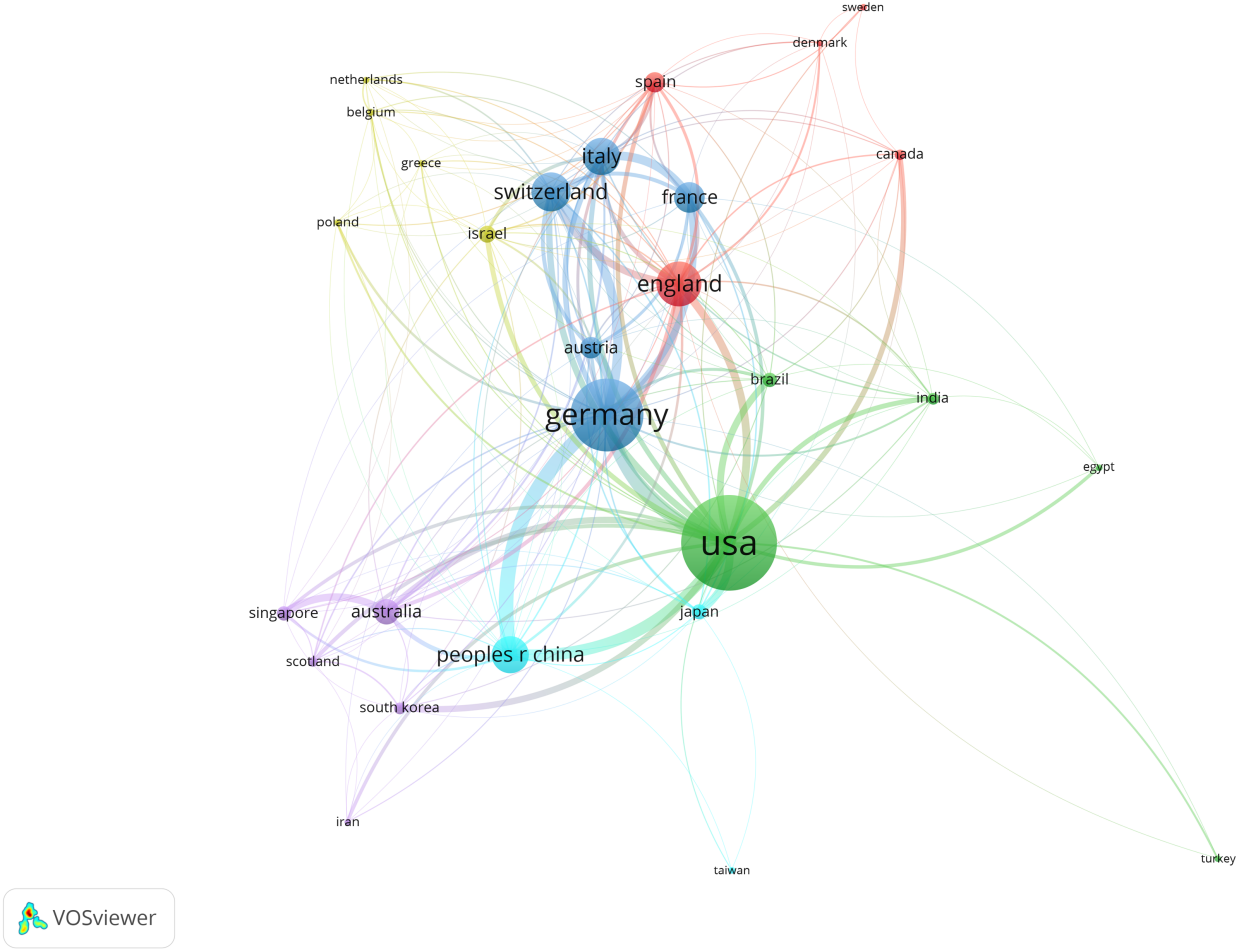
Distribution of main research countries in RVO study. The minimum number of documents of a country was set as five. Of the 75 countries that were involved in RVO research, 44 countries met the threshold.

Country co-authorship analysis reflects the degree of communication between countries as well as the influential countries in this field. The bigger nodes represent the more influential countries in this field; the thickness and distance of links between nodes represent the cooperative relationships among countries. Figure 2 showed that the United States cooperated with many countries in RVO field intensively, such as Germany, England, Japan, China and Australia. This indicates that geographical distance is not the primary influencing factor of cooperation relationships.

### Distribution of main research organizations

**Figure 3:**
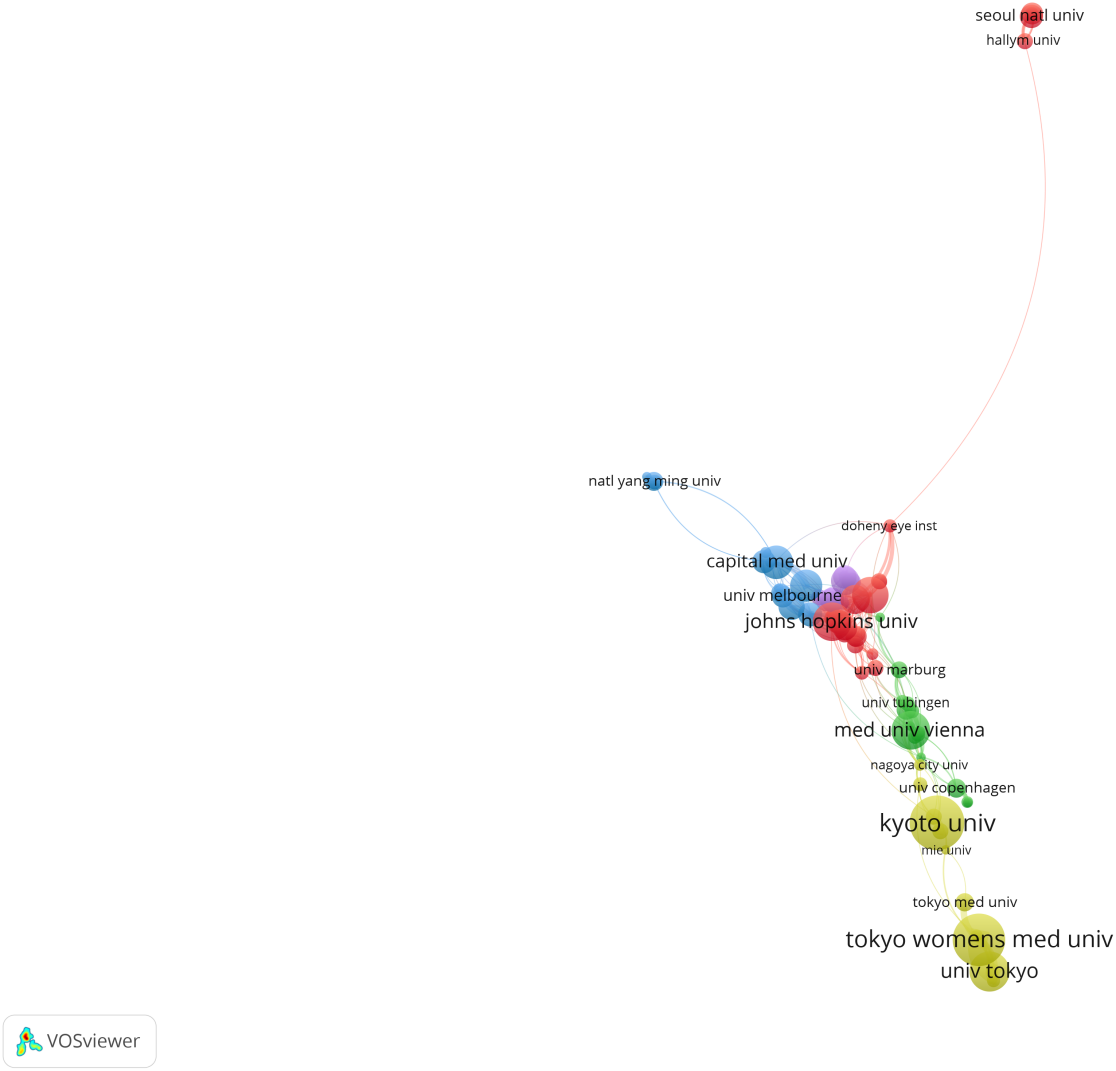
Collaboration network of main research organizations in RVO study. The minimum number of documents of an organization was set as 10. Of the 2,096 organizations that were involved in RVO research, 85 organizations met the threshold.

According to the retrieved results, the 2,135 articles were published by 2,096 organizations. The top 10 organizations have published 380 articles, accounting for 17.8% of the total number of publications (Table 2). Based on co-authorship analysis, Fig. 3 displayed the knowledge domain map of research organizations’ distribution in RVO research. The size of node corresponds to the number of published articles. The links between nodes represent the collaborations. The greater the link strength, the closer the collaboration.

### Distribution of authors and co-authorship of research groups

According to the retrieved results, over 7,497 authors contributed to RVO research. Among all authors, Noma H (54 publications) ranked the first, followed by Mimura T (51 publications) and Tsujikawa A (43 publications), indicating their productive contribution to the RVO study. The information of author co-citations was analyzed as well. Among all co-cited authors, Campochiaro Pa (1,991 co-citations) ranked first, followed by Haller JA (1,632 co-citations) and Rubio RG (1,476 co-citations), indicating their relative influence in RVO research (Table 3).

**Table 2:** Top 10 productive organizations in RVO study, 2009–2018.

Rank	Organization	Country	Count (%)	Citations
1	Kyoto University	Japan	54 (2.5)	919
2	Tokyo Women’s Medical University	Japan	52 (2.5)	645
3	University of Tokyo	Japan	40 (1.9)	549
4	Johns Hopkins University	USA	38 (1.8)	1,494
5	Medical University of Vienna	Austria	38 (1.8)	997
6	University of California Los Angeles	USA	36 (1.7)	523
7	Capital Medical University	China	33 (1.5)	319
8	Heidelberg University	Germany	32 (1.5)	544
9	University of Southern California	USA	29 (1.4)	660
10	University of Wisconsin-Milwaukee	USA	28 (1.3)	543

**Notes.**

Percentages (%) were calculated by dividing the row count by the total number of publications (*n* = 2,135).

USA United States of America

**Table 3:** Top 10 productive authors and co-cited authors in RVO study, 2009–2018.

Rank	Author	Count	Co-cited author	Count
1	Noma, H	54	Campochiaro, PA	1,991
2	Mimura, T	51	Haller, JA	1,632
3	Tsujikawa, A	43	Rubio, RG	1,476
4	Yoshimura, N	42	Bandello, F	1,219
5	Funatsu, H	31	Brown, DM	1,192
6	Jonas, JB	31	Whitcup, SM	1,149
7	Murakami, T	31	Scott, IU	1,084
8	Muraoka, Y	30	Loewenstein, A	1,046
9	Bandello, F	29	Ip, MS	932
10	Schmidt-Erfurth U	26	Yoshimura, N	914

**Figure 4:**
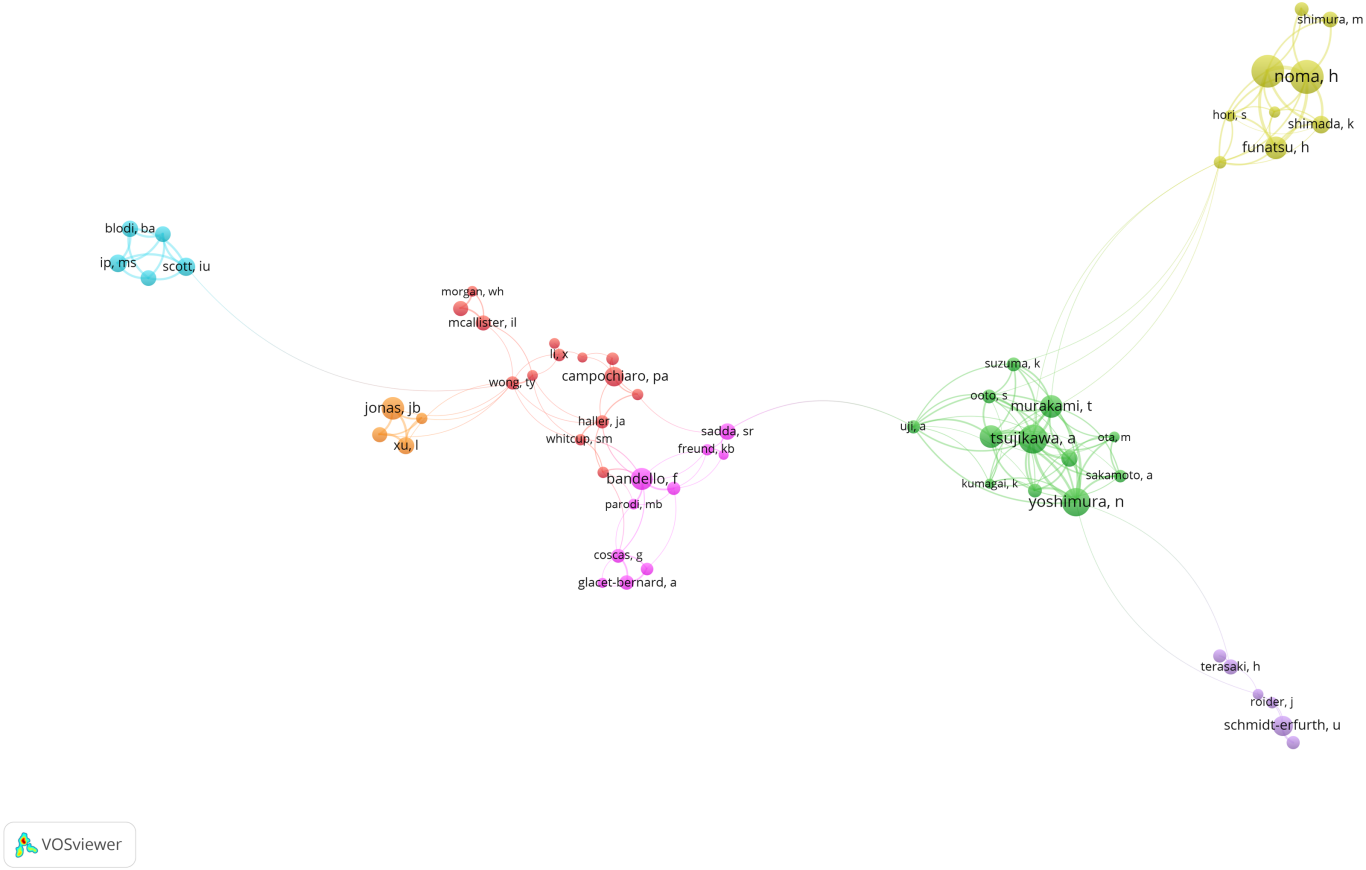
Co-authorship network of productive authors in RVO study. The minimum number of documents of an author was set as 10. Of the 7,167 authors that were involved in RVO research, 83 authors met the threshold.

Based on co-authorship analysis, Fig. 4 displayed the knowledge domain map of research groups’ distribution in RVO research. The size of node corresponds to the number of published articles. The links between nodes represent the cooperative relationship between authors. The greater the link strength, the higher density the cooperation.

### Distribution of source journals

Based on the retrieved results, articles on RVO research were distributed in 299 journals. The top 10 journals that publish on this topic are listed in Table 4. *Retina-The Journal of Retinal and Vitreous Diseases* published the highest number of articles (208, 9.7%), followed by *Investigative Ophthalmology & Visual Science* (131, 6.1%) and *Ophthalmology* (109, 5.1%). Articles published in these three journals accounted for 20.9% of all publications included in this study.

**Table 4:** Top 10 main source journals in RVO study, 2009–2018.

Rank	Journal	Country	Count	% of 2,135
1	Retina-the Journal of Retinal and Vitreous Diseases	United States	208	9.7
2	Investigative Ophthalmology & Visual Science	United States	131	6.1
3	Ophthalmology	United States	109	5.1
4	Graefes Archive for Clinical and Experimental Ophthalmology	United States	102	4.8
5	American Journal of Ophthalmology	United States	79	3.7
6	Acta Ophthalmologica	Den Mark	75	3.5
7	British Journal of Ophthalmology	England	67	3.1
8	PLOS ONE	United States	64	3.0
9	Ophthalmologica	Switzerland	60	2.8
10	European Journal of Ophthalmology	Italy	55	2.6

### Distribution of cited references: knowledge bases of RVO study

Through co-citation analysis of cited references, the intellectual base of RVO study can be constituted efficiently. The minimum citation number of a cited reference was set as 20. Of the 32,606 cited references, 305 cited references met the threshold. The top 10 cited references were presented in Table 5.

**Table 5:** Top 10 co-cited references in RVO research, 2009–2018.

Rank	Title	Cluster	Citations
1	The prevalence of retinal vein occlusion: pooled data from population studies from the United States, Europe, Asia, and Australia.	1	266
2	Ranibizumab for macular edema following branch retinal vein occlusion: six-month primary end point results of a phase III study.	4	233
3	Randomized, sham-controlled trial of dexamethasone intravitreal implant in patients with macular edema due to retinal vein occlusion.	6	214
4	Ranibizumab for macular edema following central retinal vein occlusion: six-month primary end point results of a phase III study.	4	211
5	Dexamethasone intravitreal implant in patients with macular edema related to branch or central retinal vein occlusion twelve-month study results.	6	175
6	A randomized trial comparing the efficacy and safety of intravitreal triamcinolone with standard care to treat vision loss associated with macular Edema secondary to branch retinal vein occlusion: the Standard Care vs Corticosteroid for Retinal Vein Occlusion (SCORE) study report 6.	2	174
7	A randomized trial comparing the efficacy and safety of intravitreal triamcinolone with observation to treat vision loss associated with macular edema secondary to central retinal vein occlusion: the Standard Care vs Corticosteroid for Retinal Vein Occlusion (SCORE) study report 5.	5	165
8	The epidemiology of retinal vein occlusion: the Beaver Dam Eye Study.	1	157
9	Sustained benefits from ranibizumab for macular edema following branch retinal vein occlusion: 12-month outcomes of a phase III study	4	153
10	Natural history and clinical management of central retinal vein occlusion. The Central Vein Occlusion Study Group	5	153

**Figure 5:**
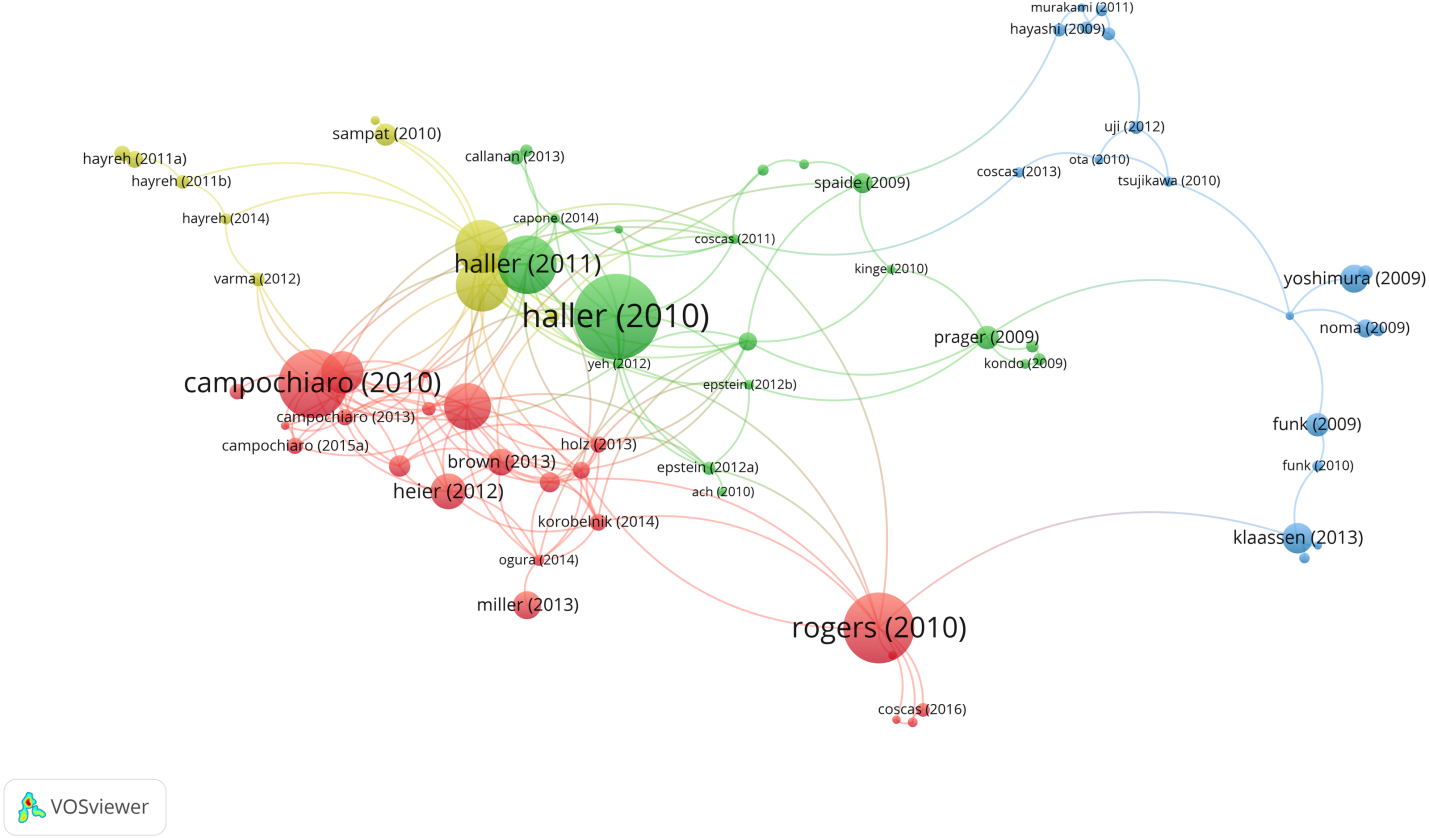
Citation analysis of documents. The minimum number of citations of a document was set as 50. Of the 2,135 documents, 103 documents met the threshold.

The minimum number of citations of a document was set as 50; through citation analysis of the 2,135 documents, 103 documents met the threshold (Fig. 5). The size of nodes corresponds to the number of citations.

**Figure 6:**
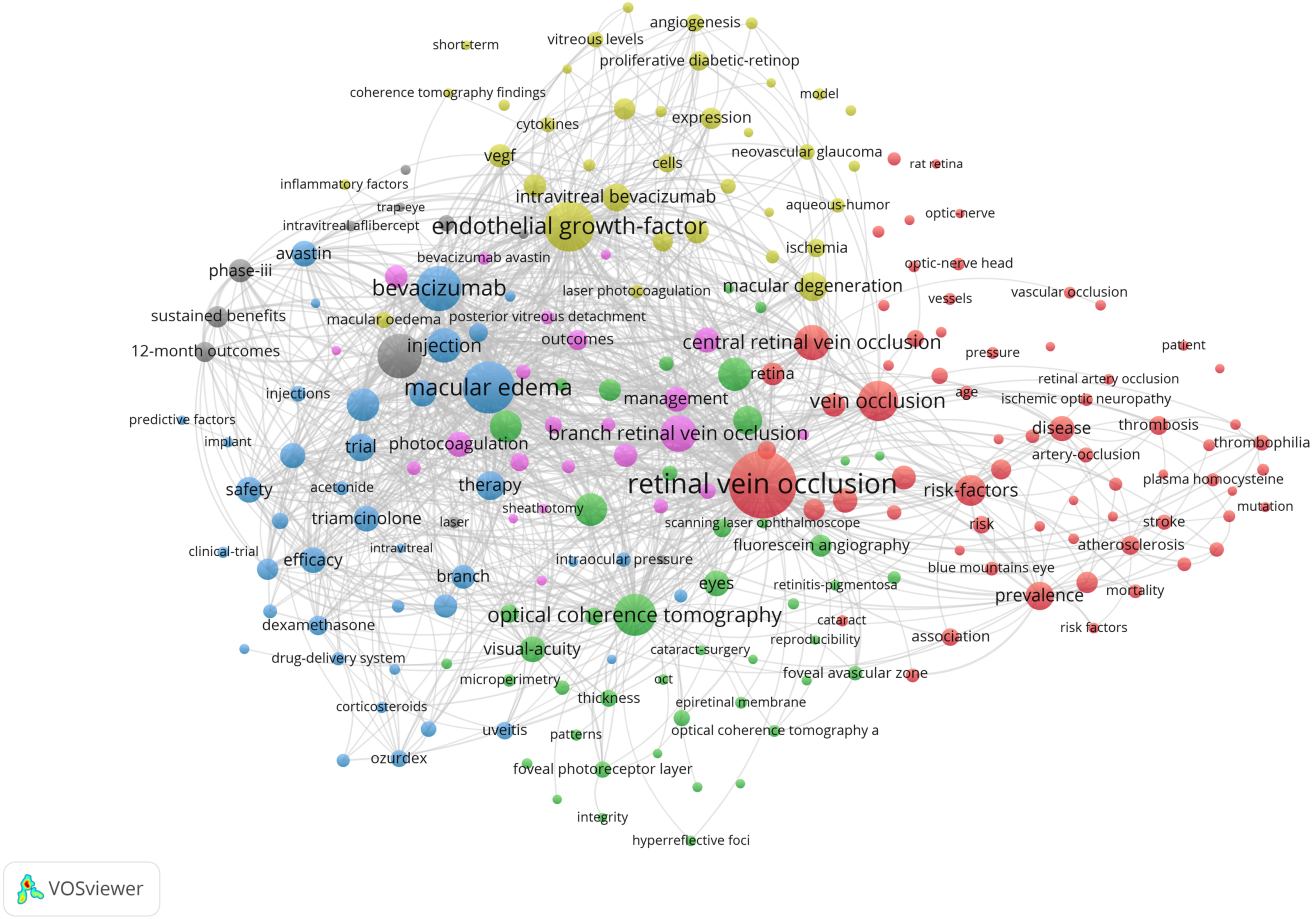
Co-occurrence network of keywords in RVO study. The minimum number of occurrences of a keyword was set as 15. Of the 5,245 keywords that were involved in RVO research, 222 keywords met the threshold.

### Distribution of keywords: hotspots of RVO study

Through co-occurrence analysis of high-frequency keywords, the research hotspots of RVO were identified. The minimum co-occurrence number of a keyword was set as 15. Of the extracted 5,245 keywords that involved in RVO, 222 keywords met the threshold. On the basis of the network, the keywords with similarities were clustered, and the six main clusters were denoted using the colors red, green, pink, blue, yellow, and grey, respectively (Fig. 6). The top 10 keywords for each cluster were listed in Table 6.

## Discussion

### Global trends in research on RVO

The quantity variation of academic papers is an important research index, which can reflect the development trend of this field. As shown in Fig. 1, a total of 2,135 papers were retrieved on RVO from 2009 to 2018, and the annual research output increased with time.

In the analysis of the most productive countries shown in Table 1, the United States accounted for 24.3% of publications and ranked the highest number of publications. This indicates that the United States is the international scientific center in RVO research.

Through the analysis of the distribution of research organizations, the most productive organizations and cooperation within the groups in a certain field can be identified. As shown in Fig. 3, University of California Los Angeles presented the highest number (46 links), followed by Johns Hopkins University (39 links) and Stanford University (34 links) indicating that these research organizations are at the core of the entire research network.

The establishment of co-authorship network knowledge map can provide possible cooperation opportunities for researchers. As shown in Fig. 4, the red-colored group has Prof. Campochiaro, PA as the center; the green-colored group has Prof. Yoshimura, N as the center; the pink-colored group has Prof. Bandello, F as the center; the yellow-colored group has Prof. Noma, H as the center; the purple-colored group has Prof. Schmidt-Erfurth, U as the center; the blue-colored group has Prof. Scott, IU as the center; the orange-colored group has Prof. Jonas, JB as the center.

A distribution analysis of academic journals helps determine the core journals in the certain field. To this end, *Retina-The Journal of Retinal and Vitreous Diseases,* which has published the highest number of articles, is the most prolific journal publishing RVO research.

**Table 6:** Co-occurence analysis of keywords. Top 10 keywords in the 6 clusters.

Cluster 1 (red)	Cluster 2 (green)	Cluster 3 (blue)	Cluster 4 (Yellow)	Cluster 5 (pink)	Cluster 6 (grey)
Retinal vein occlusion (750)	Optical coherence tomography (291)	Macular edema (438)	Endothelial growth-factor (412)	Branch retinal vein occlusion (216)	Ranibizumab (328)
Risk factors (157)	Diatebic retinopathy (184)	Bevacizumab (332)	Macular degeneration (136)	Photocoagulation (106)	phase III (87)
Prevalence (132)	Diabetic macular edema (175)	Injection (192)	Intravitreal bevacizumab (125)	Management (104)	Sustained benefits (74)
Glaucoma (92)	Fluorescein angiography (80)	Triamcinolone acetonide (103)	Neovascularization (91)	Vitrectomy (90)	12-month outcomes (67)
Pathogenesis (90)	Thickness (48)	Anti-VEGF (58)	Interleukin-6 (84)	Outcomes (67)	Aflibercept (45)
Atherosclerosis (58)	Nonperfusion (39)	Uveitis (53)	Angiogenesis (51)	Intravitreal triamcinolone acetonide (56)	Laser (23)
Thrombophilia (41)	Detachment (36)	Dexamethasone implant (41)	Cytokine (37)	Complications (41)	Intravitreal aflibercept (20)
Hypertension (31)	Foveal avascular zone (36)	Intraocular pressure (36)	Aqueous-humor (35)	Endophthalmitis (33)	Intravitreal ranibizumab (18)
Age (29)	Diagnosis (22)	Inflammation (30)	Inflammatory factors (19)	Posterior vitreous detachment (32)	Quality-of-life (16)
Oxidative stress (15)	Optical coherence tomography angiography (25)	Corticosteroids (22)	Vascular permeability (15)	Grid laser treatment (16)	Trap-eye (15)

**Notes.**

The numbers in brackets represent the frequency of keywords according to the co-occurrence analysis.

### Intellectual base

Through Co-Citation Analysis, a large number of cited references can effectively show the background of the study. Therefore, we conducted cluster analysis to explore the main topics of RVO research. As shown in Table 5, the top 10 cited references contain various clinical trials which mainly presented anti-VEGF medications on the management of RVO. Publications entitled “The prevalence of retinal vein occlusion: pooled data from population studies from the United States, Europe, Asia, and Australia”. and “Ranibizumab for macular edema following branch retinal vein occlusion: six-month primary end point results of a phase III study”. ranked the top two in both frequency count and link weight, respectively, which are considered in the core position of the whole knowledge map. In spite of not belonging to the top 10 cited references, publication entitled “Vascular endothelial growth factor in ocular fluid of patients with diabetic retinopathy and other retinal disorders” mainly reported intraocular concentrations of VEGF correlated with active neovascularization. This paper ranked the third in link weight, which demonstrates this finding plays an important role in the knowledge map structure.

### Research frontiers

The co-occurrence of keywords is supposed to represent the searching theme. Thus, the internal structure of the related literature and the frontier discipline can be revealed. As shown in Fig. 6, the topics of RVO mainly formed six clusters, and the keywords with similarity in research topics are grouped together. Combined with the characteristics and current status of RVO study, the 6 clusters were analyzed as follows:

Cluster #1 (red colored) represented keywords mainly related to the risk factors and pathogenesis of RVO. The prevalence of RVO has been reported to range between 0.4% and 4.6%. Of the two main types of RVO, BRVO is 4–6 times more prevalent than CRVO (Kolar, 2014). Age and systemic diseases such as atherosclerosis were the most common risk factors for RVO. The prevalence of RVO increases significantly with age, but not with gender (Rogers et al., 2010). Systemic diseases such as hypertension, diabetes mellitus, thrombophilic diseases like factor-V-Leiden mutation and hyperhomocysteinemia are associated with RVO (Jaulim et al., 2013; Nema, Verma & Kumar, 2018). It has been reported in the literature that there is a correlation between RVO and glaucoma. After 11 years follow-up, Park et al. (2017) reported a significantly higher risk of glaucoma in RVO patients. This can be explained by the fact that RVO and glaucoma have some risk factors in common (Fraenkl, Mozaffarieh & Flammer, 2010). Primary angle closure glaucoma (PACG) resulted in mechanical changes in lamina cribrosa of the optic disc and the increase of cup/disk ratio was considered as an important risk factor for RVO (Xu et al., 2018). RVO is characterized by high blood viscosity, but the pathogenesis is still unclear. Becatti et al. (2016) compared the production of reactive oxygen species (ROS) and membrane lipid peroxidation in RVO patients and control groups. The results showed that erythrocyte ROS stress was positively correlated with blood viscosity and played a key role in the pathogenesis of RVO. (Becatti et al., 2016).

Cluster #2 (green colored) represented keywords related to diagnostic methods of RVO. OCT measurement often plays vital role in the diagnosis of fundus diseases. Due to the high-resolution imaging performance, OCT helps detect the presence of macular edema, vitreoretinal interface changes, retinal detachment and subretinal fluid (Noma et al., 2011). OCTA is able to assess retinal hemodynamics in RVO patients, and microvascular changes in both the superficial and deep capillary networks of the retina are visible (Tsai et al., 2018). FFA can detect peripheral capillary nonperfusion area, macular ischemia and neovascularization. The more capillary nonperfusion regions are, the greater risk of angiogenesis is. FFA can also help distinguish collateralization from neovascularization, since the former does not leak fluoresence, whereas the latter does (Ip & Hendrick, 2018).

Cluster #3 (blue colored) represented keywords related to the therapeutic use of corticosteroids on RVO. Cystoid macular edema (CME) is caused by congestion of capillaries and is characterized by metamorphopsia and loss of visual acuity. Anti-VEGF injections can benefit most patients. However, the ability of anti-VEGF injections to reduce intraretinal fluid becomes less pronounced in these patients with the passage of time (Campochiaro et al., 2015). Dexamethasone implants have a longer duration of action in reducing chronic or recurrent edema. It is a beneficial alternative for patients who still have residual edema after anti-VEGF injection or need regular injection to control edema (Boyer et al., 2014). Singer et al. (2012) compared the efficacy and duration effect applying combination therapy of bevacizumab and dexamethasone with either of the medication. Combined therapy has the advantages of synergistic effect, improving visual acuity and prolonging injection intervals (Singer et al., 2012). Inflammation is also involved in the pathogenesis of macular edema after RVO and noninfectious posterior segment uveitis (NIPSU) (Tufail et al., 2018). Studies have shown that aqueous humor levels of proinflammatory cytokines are reduced after DEX treatment in patients with RVO (Rezar-Dreindl et al., 2017).

Cluster #4 (yellow colored) represented keywords related to the metabolism of RVO. Several ophthalmic diseases, including DR, macular degeneration and macular edema secondary to RVO, are characterized by increased vascular permeability and abnormal angiogenesis in retina (Avery et al., 2017). High retinal vascular permeability and blood-retinal barrier (BRB) damage are important in the pathophysiology of BRVO related macular edema (Noma, Mimura & Eguchi, 2013). Macular edema secondary to RVO is associated with the rising level of VEGF in the aqueous humor. VEGF is a key intermediary of physiological and pathological angiogenesis. Angiogenesis and inflammatory cytokines in ocular fluid have been implicated in endothelial cell injury. Endothelial cells exposed to proinflammatory cytokines may induce oxidative stress and cell apoptosis (Funatsu et al., 2012). Concentrations of IL-1*α*, -6, and -8; IP-10; and PDGF-AA were significantly increased in RVO patients compared with control group (Funk et al., 2009). Therefore, the management of macular edema secondary to RVO should pay attention to the concentration of VEGF and inflammatory cytokines (Machalinska et al., 2016).

Cluster #5 (pink colored) represented keywords related to the management of macular edema secondary to RVO. Patients with RVO are at risk of multiple complications, and macular edema is the most common cause of the visual impairment (Rehak & Wiedemann, 2010). Macular grid photocoagulation is an effective method for the treatment of macular edema in patients with BRVO. Other treatments for edema include intravitreal injection of corticosteroids, anti-VEGF drugs and vitrectomy. Studies have shown that vitrectomy can effectively reduce macular edema and improve visual acuity in BRVO patients. The mechanism may be that oxygen-containing liquid circulates in the vitreous cavity may improve the perifoveal microcirculation, thereby increasing the clearance of VEGF in the vitreous cavity (Nishida et al., 2017). Scott et al. (2009) studied the efficacy and safety of IVTA at 1-mg/4 mg dose and standard grid photocoagulation in the treatment of BRVO. OCT thickness was improved to varying degrees in all groups during the 1-year observation period. In terms of complications, cataract progression rate was higher in the 4 mg IVTA group, thus IVTA is less commonly used than anti-VEGF therapy (Scott et al., 2009).

Cluster #6 (grey colored) represented keywords related to the anti-VEGF treatment of RVO. Currently, three anti-VEGF agents are used routinely for the treatment of RVO; two are FDA-approved (Ranibizumab and Aflibercept), but bevacizumab remains off-label for ophthalmologic conditions. These three anti-VEGF treatments differ in their molecular structure and properties. Ranibizumab is an anti-VEGF-A monoclonal antibody fragment which does not contain the Fc antibody region, and hence, it has a short systemic elimination time (Ferrara et al., 2006). Campochiaro et al. (2011) reported the six-month sustained benefits from Ranibizumab treatment for macular edema following CRVO. Aflibercept is a fusion protein that binds multiple isoforms of human VEGF-A, VEGF-B and placental growth factor (Heier et al., 2014). Various clinical trials have been conducted to determine the optimum drug, elucidate the efficacy and guide administration frequency of the three anti-VEGF treatments in patients with AMD, DME and RVO.

## Conclusion

We constructed a series of science maps of the annual publication number, the distribution of countries, international collaborations, author productivity, source journals, cited reference and keywords in RVO study. The results of this study may be helpful for ophthalmologists in choosing appropriate journals for publication and organizations or authors for collaborations. The extracted keywords enable researchers to identify new topics, and assist them in predicting research directions. However, there are some limitations that should be considered. First, the publications were extracted from WoSCC between 2009 and 2018, which may not be enough to represent all of the topics in RVO research. Second, the primary data were extracted from WoSCC, which is a database more suited for performing citation analysis. Third, because most publications in the WoSCC were in English, a linguistic bias may exist. Last but not the least, the collaboration network analysis successfully displays the co-occurrence (distance between the two nodes/items) and the co-authorship of the institutions (the strength of the links). However, neither is the strength of each linked two items shown in the final exported file, nor can a geological map with co-authorship be generated by VOSviewer. Thus, a visualization of geological location and co-authorship cannot be generated, and an understanding of the relationship thereof cannot be calculated.

##  Supplemental Information

10.7717/peerj.7603/supp-1 Supplemental Information 1 Cited reference for RVO study, 2009–2018 The minimum number of citations of a cited reference was set as 20. Of the 32,606 cited references that involved in RVO research, 305 cited references meet the threshold.

## Additional Information and Declarations

### Data Availability

The following information was supplied regarding data availability: The raw data is available in the Supplemental Files.
